# Extension of Public Smoking Ban Was Not Associated with Any Immediate Effect on Stroke Occurrence in Finland

**DOI:** 10.3390/jcm10102060

**Published:** 2021-05-11

**Authors:** Jussi O. T. Sipilä, Jori O. Ruuskanen, Jussi P. Posti, Päivi Rautava, Ville Kytö

**Affiliations:** 1 Department of Neurology, North Karelia Central Hospital, Siun Sote, 80210 Joensuu, Finland; 2 Clinical Neurosciences, University of Turku, 20520 Turku, Finland; jori.ruuskanen@tyks.fi; 3 Department of Neurology, Neurocenter, Turku University Hospital, University of Turku, 20521 Turku, Finland; 4 Department of Neurosurgery and Turku Brain Injury Centre, Neurocenter, Turku University Hospital, University of Turku, 20521 Turku, Finland; jussi.posti@utu.fi; 5 Department of Public Health, Turku Clinical Research Centre and Turku University Hospital, University of Turku, 20521 Turku, Finland; paivi.rautava@tyks.fi; 6 Heart Center, Turku University Hospital, 20521 Turku, Finland; vijoky@utu.fi; 7 Research Center of Applied and Preventive Cardiovascular Medicine, University of Turku, 20520 Turku, Finland; 8 Center for Population Health Research, Turku University Hospital, University of Turku, 20521 Turku, Finland; 9 Administrative Center, Hospital District of Southwest Finland, 20521 Turku, Finland

**Keywords:** public health, smoking, smoking ban, stroke

## Abstract

We investigated the association between the widening of a nationwide restaurant smoking ban, enacted on 1 June 2007, and stroke admissions. All acute stroke admissions between 1 May 2005 and 30 June 2009 were retrieved from a mandatory registry covering mainland Finland. Patients aged ≥18 years were included. One annual admission per patient was included. Negative binomial regression accounting for the at-risk population was applied. We found no difference in stroke occurrence before and after the smoking ban within 7 days (*p* = 0.217), 30 days (*p* = 0.176), or the whole study period (*p* = 0.998). Results were comparable for all stroke subtypes (ischemic stroke, intracerebral hemorrhage, and subarachnoid hemorrhage). There was no sign of decreased occurrence in June 2007 compared to June in 2005–2006, and all subtypes of stroke occurred at least as frequently in both May and June of 2008 as in May and June of 2007. In conclusion, the nationwide restaurant smoking ban Finland enacted in June 2007 was not associated with any immediate reduction in stroke occurrence.

## 1. Introduction

Smoking is a strong risk factor for cardiovascular disease, including stroke, and secondhand smoke (SHS) also increases stroke risk [1]. Many countries and regions across the world have implemented public smoking bans to improve public health, and these seem effective in reducing myocardial infarctions [2,3]. However, data on stroke occurrence in this setting are more limited [3,4,5]. Previous data showed that smoking causes subarachnoid hemorrhage (SAH), the incidence of which has steadily declined in Finland along with decreasing smoking prevalence [6,7].

A nationwide workplace smoking ban in Finland was enacted in 1995 and extended to cover restaurant workers in 2000. The latest extension came on 1 June 2007, when smoking was prohibited indoors in all public places (a transition time until June 2009 was offered for large restaurants, but only 5% used this opportunity). This extension was associated with reduced rates of smoking, SHS exposure, and myocardial infarction (MI) [8,9]. We investigated if there was also a change in stroke occurrence.

## 2. Methods

All admissions to neurological, neurosurgical, and intensive care wards with acute stroke (ICD-10 codes I60.X-I63.X) as the primary diagnosis between 1 May 2005 and 30 June 2009 were identified from the Care Register for Health Care (CRHC), a mandatory database for all public health care hospital discharges in Finland. All hospitals that provide acute stroke care in mainland Finland were included. Only one annual admission per patient was included. Patients under 18 years of age and patients with concurrent ICD-10 diagnosis codes of rehabilitation (Z50.X) were excluded. Occurrence of stroke admissions was analyzed with count regression. Analysis of the whole study period was adjusted for month to account for seasonality in stroke occurrence. Due to overdispersion, negative binomial modeling was applied. The logarithm of population at risk for each calendar year (total number of 20,923,720 person-years) was used as an offset parameter. Stroke occurrence before and after the restaurant smoking ban within and 7 and 30 days and the whole study period was studied.

Furthermore, *p*-values of less than 0.05 were considered significant. IBM SPSS Statistics version 26 (IBM SPSS, Chicago, IL, USA) and SAS version 9.4 (SAS Institute, Cary, NC, USA) were used for statistical analyses. Since stroke occurrence is seasonal and the ban was instituted at the beginning of June, a separate visualization of only May and June admissions was also performed [10]. Being the days when restaurants, pubs, and nightclubs are most often visited, Friday and Saturday admissions in May and June were also investigated separately.

This study was approved by the Finnish Institute for Health and Welfare (THL, permission no: THL/2245/5.05.00/2019). This was a retrospective register study, and thus, no informed consent was required, and the participants were not contacted.

## 3. Results

**Figure 1:**
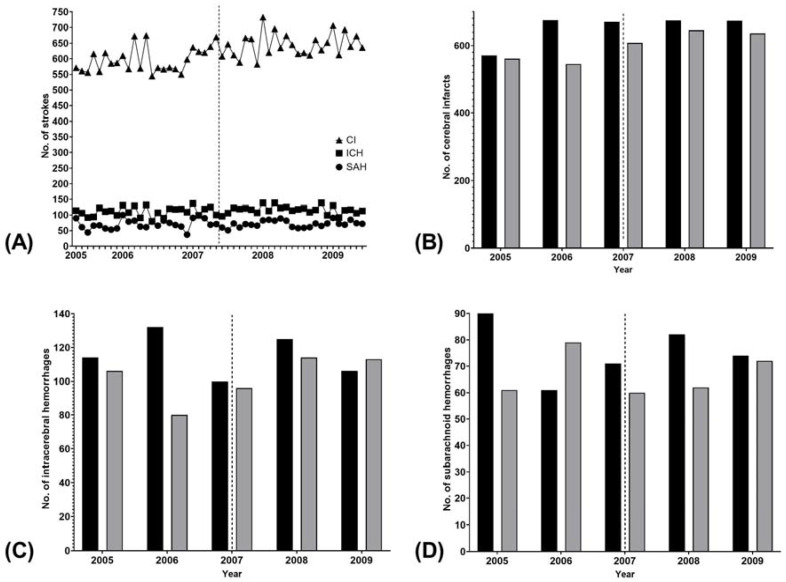
Number (N) of stroke admissions by stroke type. (**A**) All admissions from May 2005 to June 2009; (**B**) May and June admissions for cerebral infarction; (**C**) May and June admissions for intracerebral hemorrhage; (**D**) May and June admissions for subarachnoid hemorrhage. The dashed line marks the introduction of the nationwide restaurant smoking ban on 1 June 2007. CI, cerebral infarction; ICH, intracerebral hemorrhage; SAH, subarachnoid hemorrhage. Black bars, May; grey bars, June.

There were 31,023 admissions for cerebral infarction, 5680 admissions for intracerebral hemorrhage (ICH), and 3555 admissions for SAH during the study period (Figure 1A). There was no difference in stroke occurrence before and after the smoking ban within 7 days (*p* = 0.217), 30 days (*p* = 0.176), or the whole study period (*p* = 0.998) (Table 1). The results were consistent in all stroke subtypes (*p* ≥ 0.102 for all) (Table 1). The visual inspection revealed a trend for lower numbers of all stroke types in June 2007 compared to May 2007, but this pattern did not differ from the years preceding and following the smoking ban (Figure 1B–D). Moreover, all strokes occurred at least as frequently in both May and June of 2008 as in the respective months in 2007.

No difference in Friday or Saturday stroke admissions before vs. after the smoking ban was observed within the study period (*p* = 0.101). Compared to May 2007, June admissions were indeed slightly more numerous on Fridays and Saturdays for all stroke types in June 2007 (data not shown).

## 4. Discussion

These data show no clear reduction in any type of acute stroke associated with the nationwide restaurant smoking ban enacted in Finland. Previous studies on the subject have usually reported decreased stroke admissions after smoking ban implementations [3]. On the other hand, although admissions for MI decreased in both New York, United States, and Geneva, Switzerland, there was no change in stroke occurrence in either [5,11]. These differences may be related to differences in the study methodologies, background stroke risk and occurrence rates as well as smoking prevalence. Moreover, in Ontario, Canada, the existence and extent of previous smoking bans affected the results [12]. Finland had already enacted a limited public smoking ban in 1995 and tightened it in 2000 (see above). In comparison, only some areas in Arizona, for example, had a smoking ban of one kind or another in place before the introduction of a statewide ban [4]. It seems that the effect smoking bans have on stroke occurrence depends on their extent and the environment in which they are enacted.

The restaurant smoking ban in Finland has previously been associated with decreases in hospitalizations and in-hospital mortality for MI [8]. The fact that this study showed no immediate association between the ban and stroke hospitalizations is probably related to the fact that smoking raises the risk of MI more than it does the risk of stroke. In addition, smoking cessation also lowers the risk of MI more than that of stroke [13,14]. Moreover, patients with ischemic stroke and ICH are older than those with MI in Finland and, thus, are unlikely to visit areas covered by the ban as frequently [8,10]. However, as SHS has been shown to be an independent risk factor for stroke, reductions in stroke occurrence are also likely to occur in the long term after a widespread reduction in SHS exposure and are by no means excluded by the current results [1].

Naturally, since this is a retrospective registry study, there are caveats concerning the validity of the data. However, the registry is reliable concerning primary diagnoses, which we used here, and there were no relevant changes in treatment or coding practices during the study period [15]. Confounding by unmeasured factors such as climate and economic and social factors remains possible and should be considered when comparing the results obtained elsewhere. Lastly, incidence was only estimated roughly because of the unavailability of monthly population size and composition. However, since the size of the population >18 years of age increased by only 2.6% in Finland during the study period, with only a marginal change in the age structure, and since it seems unlikely that there was any meaningful change between May and June 2007, a more exact analysis would probably have yielded similar results. Thus, if the ban would have had a large effect, it should have been observable between these months, although the inter-year variability may have masked a moderate effect.

In conclusion, despite its effect on MI hospitalizations, the nationwide restaurant smoking ban Finland was not associated with any immediately observable effects on stroke occurrence.

## Figures and Tables

**Table 1:** Occurrence of stroke before and after restaurant smoking ban within 7 days, 30 days, and whole study period (761 days before and after the ban). Rate ratio (RR) comparing after vs. before the ban.

	Number			
Time Period	Before	After	RR (95% CI)	*p*-Value
**7 days**				
Total	209	184	0.88 (0.72–1.07)	0.217
Ischemic stroke	176	153	0.87 (0.70–1.08)	0.205
Intracerebral hemorrhage	22	15	0.68 (0.35–1.31)	0.248
Subarachnoid hemorrhage	11	16	1.09 (0.59–2.03)	0.335
**30 days**				
Total	820	764	0.93 (0.84–1.03)	0.176
Ischemic stroke	653	608	0.93 (0.83–1.05)	0.248
Intracerebral hemorrhage	98	96	0.98 (0.74–1.30)	0.886
Subarachnoid hemorrhage	69	60	0.87 (0.62–1.23)	0.428
**Study period**				
Total	19,441	20,817	1.03 (0.51–2.08)	0.998
Ischemic stroke	14,906	16,117	1.03 (0.98–1.09)	0.234
Intracerebral hemorrhage	2765	2915	1.05 (0.97–1.12)	0.102
Subarachnoid hemorrhage	1770	1785	1.00 (0.93–1.07)	0.998

## Data Availability

This manuscript is based on third-party data. Access to data is regulated by Finnish law and THL. The disclosure of data to third parties without explicit permission from THL is prohibited. Only those fulfilling the requirements established by Finnish law and THL for viewing confidential data can access the data. We confirm that the authors did not have any special access privileges that others would not have. The legal basis for processing personal data is public interest and scientific research (EU General Data Protection Regulation 2016/679 (GDPR), Article 6(1)(e) and Article 9(2)(j); Data Protection Act, Sections 4 and 6).
